# Case of Excision of the Scapula

**Published:** 1854-07

**Authors:** 


*Case of Excision of the Scapula.* By Professor Gross, of Louisville.

The disease in this case was an osteo-sarcomatous mass, measuring fifteen inches in all directions, implicating the whole of the scapula, growing rapidly, and wearing down the strength of the patient by the pain it occasioned. The patient was a slim and delicate man, aged forty.

A full dose of chloroform having been administered, an incision, sixteen inches in length, was made from the superior angle of the scapula to the inferior extremity of the tumor, its direction being obliquely downward and inward. Another, beginning about five inches below the upper end of the first, and terminating about the same distance from its lower end, was then carried, in a curvilinear direction, so as to include the small oval flap of skin with the tubercle, previously alluded to, in its center. The integuments, which were exceedingly dense and thick, especially at the superior part of the tumor, were then dissected off from the surface of the morbid growth, first toward the spine, and then toward the axilla. Having detached the elevator and trapezius muscles, I sawed through the acromion process of the scapula just behind the clavicle, and then divided the broad dorsal and anterior serrated muscles. Carrying my fingers next underneath the tumor, and raising it up, I severed its connections with the ribs, cut the deltoid and other muscles of the arm, sawed the neck of the scapula, and thus removed the entire mass with comparatively little difficulty.

Several vessels were divided in the early stage of the operation, at the posterior and middle part of the tumor; but these were easily controlled by the fingers of my assistants. Several arteries near the neck of the bone bled so freely as to demand the ligature after the removal of the morbid growth. About twenty-four ounces of blood were lost. The patient became very faint toward the close of the operation, and cordials were necessary to revive him. The immense wound thus produced was dressed with three interrupted sutures and adhesive straps, and supported by a compress and a broad body bandage. The patient was placed in bed, and immediately took one grain of morphia. At four o'clock in the afternoon there was a slight oozing of blood from the wound, and the patient complained of the tightness of the dressings, which, however, were found to be sufficiently loose. He had taken half a grain more of morphia, had slept somewhat, and was free from pain; the pulse was seventy-six, and of good volume; and there was no nausea, urgent thirst, or restlessness. On the following evening, September 27, the patient having slight traumatic fever, was ordered ten grains of calomel, with one of opium and one of ipecacuanha, to be followed in the morning by castor oil.

No untoward symptoms of any kind occurred after the operation; nearly the whole wound healed by the first intention; and at the end of three weeks, my patient went home with every prospect of a long and prosperous life. In descending the Ohio river, however, which was at that time exceedingly low, and which caused his detention upon the way for nearly a fortnight, he took a severe cold, from the effects of which he never completely recovered. A harassing cough set in, accompanied by all the symptoms of pleuro-pneumonia, which were followed, about the middle of Decemb'r, by those of hectic fever, under which he gradually sunk, three months after the operation. There was no *post-mortem* examination.—*American Quarterly Journal of Medicine.*

